# Racial Segregation, Testing Site Access, and COVID-19 Incidence Rate in Massachusetts, USA

**DOI:** 10.3390/ijerph17249528

**Published:** 2020-12-19

**Authors:** Tao Hu, Han Yue, Changzhen Wang, Bing She, Xinyue Ye, Regina Liu, Xinyan Zhu, Weihe Wendy Guan, Shuming Bao

**Affiliations:** 1 Center for Geographic Analysis, Harvard University, Cambridge, MA 02138, USA; taohu@g.harvard.edu (T.H.); wguan@cga.harvard.edu (W.W.G.); 2 Geocomputation Center for Social Science, Wuhan University, Wuhan 430079, China; 3 Center of GeoInformatics for Public Security, School of Geography and Remote Sensing, Guangzhou University, Guangzhou 510006, China; 4 Department of Geography and Anthropology, Louisiana State University, Baton Rouge, LA 70803, USA; wchang8@lsu.edu; 5 Institute for Social Research, University of Michigan, Ann Arbor, MI 48106, USA; coolnanjizhou@gmail.com; 6 Department of Landscape Architecture and Urban Planning, Texas A&M University, College Station, TX 77840, USA; xinyue.ye@tamu.edu; 7 Department of Biology, Mercer University, Macon, GA 31207, USA; Regina.liu@live.mercer.edu; 8 State Key Laboratory of Information Engineering in Surveying, Mapping and Remote Sensing, Wuhan University, Wuhan 430079, China; geozxy@263.net; 9 Collaborative Innovation Center of Geospatial Technology, Wuhan University, Wuhan 430079, China; 10 China Data Institute, Ann Arbor, MI 48108, USA; sbao@umich.edu

**Keywords:** COVID-19 incidence rate, racial segregation, access to testing site, spatial regression

## Abstract

The U.S. has merely 4% of the world population, but contains 25% of the world’s COVID-19 cases. Since the COVID-19 outbreak in the U.S., Massachusetts has been leading other states in the total number of COVID-19 cases. Racial residential segregation is a fundamental cause of racial disparities in health. Moreover, disparities of access to health care have a large impact on COVID-19 cases. Thus, this study estimates racial segregation and disparities in testing site access and employs economic, demographic, and transportation variables at the city/town level in Massachusetts. Spatial regression models are applied to evaluate the relationships between COVID-19 incidence rate and related variables. This is the first study to apply spatial analysis methods across neighborhoods in the U.S. to examine the COVID-19 incidence rate. The findings are: (1) Residential segregations of Hispanic and Non-Hispanic Black/African Americans have a significantly positive association with COVID-19 incidence rate, indicating the higher susceptibility of COVID-19 infections among minority groups. (2) Non-Hispanic Black/African Americans have the shortest drive time to testing sites, followed by Hispanic, Non-Hispanic Asians, and Non-Hispanic Whites. The drive time to testing sites is significantly negatively associated with the COVID-19 incidence rate, implying the importance of the accessibility of testing sites by all populations. (3) Poverty rate and road density are significant explanatory variables. Importantly, overcrowding represented by more than one person per room is a significant variable found to be positively associated with COVID-19 incidence rate, suggesting the effectiveness of social distancing for reducing infection. (4) Different from the findings of previous studies, the elderly population rate is not statistically significantly correlated with the incidence rate because the elderly population in Massachusetts is less distributed in the hotspot regions of COVID-19 infections. The findings in this study provide useful insights for policymakers to propose new strategies to contain the COVID-19 transmissions in Massachusetts.

## 1. Introduction

Since its outbreak in January, the COVID-19 pandemic has severely impacted socioeconomic activities throughout the world. As of 3 July, there have been 10,719,946 confirmed cases globally, including 517,337 deaths. The United States is the leading country with 2,671,220 confirmed cases and 127,858 deaths [1]. Since the beginning of April, the U.S. has become the COVID-19 pandemic center, and the number of cases is still increasing. Social distancing is one of the most effective ways to reduce COVID-19 infection, but due to residential segregation—the separation of people based on income and/or race—some individuals from specific ethnic minority groups cannot practice social distancing. They are often found in overcrowded urban housing areas, which make physical distancing and self-isolation difficult. This leads to an increased risk for the spread of COVID-19 [2]. Moreover, socioeconomic inequities frequently impact health and healthcare access, resulting in a higher burden of disease and mortality in vulnerable social groups. Therefore, it is necessary to integrate social-economic information and disease statistics to help analyze and understand the spread of COVID-19.

Many research findings have highlighted racial disparities in the transmission of COVID-19. Across the country, deaths caused by COVID-19 are disproportionately high among African Americans [3], while Chicago and New York City reported greater COVID-19 mortality among Latinos [4]. To utilize more detailed information on the racial and socioeconomic disparities, Raifman [5] used the 2018 Behavioral Risk Factor Surveillance System (BRFSS) to estimate the proportion of adults that meets at least one of the many CDC (Centers for Disease Control and Prevention) criteria for risk of severe illness from COVID-19. The analysis is categorized by age group, race, and household income. Results show that people who are Black, American Indian, or live in low-income households are more likely to have conditions associated with increased risk of illness from COVID-19, compared to those who are White or have a higher income, respectively. Anyane-Yeboa, et al. [6] investigated the racial disparity of infection and deaths caused by COVID-19 in the U.S. and assessed the rates of COVID-19 infection and death by race and ethnicity with information obtained from the Department of Health websites of sixteen states. They stated that Black patients had higher rates of infection and death from COVID-19, which are consistent with the findings from some other individual states. Laurencin and McClinton [7] presented the overview of racial and ethnic distribution of COVID-19 confirmed cases and fatalities in the state of Connecticut to demonstrate the unique challenges among Black and Brown communities. At the metropolitan level, Yu et al. [8] examined the growth rate of both COVID-19 confirmed cases and deaths in the first 30-day period of the outbreak within 100 of the largest metropolitan cities. They observed that the growth curve was particularly steep in counties that are located in cities with high economic disparity and residential segregation of Blacks and Hispanics.

One fundamental cause of racial disparities in health is racial residential segregation, which presents the physical isolation of one racial group from others [9]. This segregation can affect health through concentrated poverty, the quality of the neighborhood environment, and the individual socioeconomic attainment of minorities [10]. Health experts believe that person-to-person and community transmission are the most common ways to spread the COVID-19. Thus, neighborhoods with concentrated poverty and over-occupancy of housing units are at a higher risk of COVID-19 infection. Furthermore, minorities under the poverty line are more likely to work in industries that have remained open during non-essential business closures [5]. Thus, they have greater exposure to COVID-19 and also contribute to the transmission of COVID-19. Given the influence of racial residential segregation on the socioeconomics of neighborhoods, it is necessary to conduct a systematic investigation of whether segregation has a direct impact on COVID-19 transmission.

In addition, adequate access to affordable testing sites and hospitals is critically important to identify potential carriers of COVID-19. Since it is the best way to provide evidence-based decisions to slow down the disease, the method of ”test, test, test” has been greatly endorsed by the World Health Organization to countries around the world. Appropriate Public Health measures, such as self-isolation and hospitalization, must also be taken to contain the pandemic. Many states, with the support of the federal government, have expanded access to COVID-19 related health care services either by setting up more mobile testing sites and increasing testing providers or providing telehealth to cover as many people as possible. However, these testing sites are more likely to be distributed in the well-off suburbs of White-dominant neighborhoods rather than in low-income minority neighborhoods [11]. Such disparities in health care access are becoming worse among socioeconomically disadvantaged groups due to the lack of health insurance, access to transportation, and individual awareness of the disease severity. This is demonstrated by the fact that there has been an estimated 25% of the Black population accounting for 41% of COVID-19 cases in Boston [12]. Additionally, the Hispanic communities are reported to be at higher risk of infection and death from COVID-19. Hence, a reliable and accurate measure of access to testing sites could help us understand which areas and what demographic groups suffer from inadequate access and the best testing strategies to adopt in efforts to mitigate the COVID-19 pandemic.

According to previous studies, sociodemographic and economic, as well as environmental features, are also important factors in affecting the spread of COVID-19 disease. For example, in the analysis conducted by Mollalo et al. [13], they considered income inequality, median household income, the percentage of nurse practitioners, and the percentage of the Black female population when modeling the COVID-19 incidence at the U.S. county level. The results demonstrated that areas with a high incidence of COVID-19 usually have high-income inequality and median household income [13]. Liu [14] collected the number of laboratory-confirmed COVID-19 cases in 312 cities in China, and a series of sociodemographic variables such as distance to the epicenter, the total length of built urban metro lines, urban area, population density, the annual quantity of wastewater discharged, and residential garbage connected and transported, per capita public recreational green space, the daily highest temperature, and the capital city. Based on these data, a study of the impacts of COVID-19 transmission was conducted from the urban perspective. The statistically significant results revealed that the amount of residential garbage that was connected and transported, in conjunction with the annual quantity of wastewater discharged, could increase the confirmed infection number of COVID-19 [14].

In the midst of COVID-19 mitigation efforts, to explore regional patterns and inform local policymakers about the efficient allocation of resources and personnel, making inferences at finer scales, such as the sub-county, census tract, or block group, may produce more accurate results than coarse levels, such as the county or above. In Chicago, more than 50% of COVID-19 cases and nearly 70% of COVID-19 deaths involve Black individuals. These deaths are concentrated in just five neighborhoods in the city’s South Side. Thus, it is critical to quantify the interaction between the various factors and disease statistics at a finer scale. Analyzing regional patterns and increasing their awareness of the COVID-19 dangers and preventative measures will benefit the most affected communities [15]. Quantifying disparities in risk is important for allocating resources to prevent, identify, and treat COVID-19-related severe illness and limit diverging outcomes for vulnerable subgroups.

The spatial autocorrelation effect is an important issue when modeling geographic distributions of various events, such as crime, housing, and other human activities. Recent studies have demonstrated that health care events are also affected by spatial autocorrelation. Spatial autocorrelation is an instantiation of the first law of geography addressed by Waldo Tobler [16]. That is, all things are related, but near things are more related than distant things. It suggests the observations at different locations are not independent or that the spatial pattern is not random. In other words, it captures the association of the observed variables in one location and the neighboring areas. The closer the geographic phenomena are across space, the more similar they are. For example, high cancer mortality in one area is more likely to be bordered by neighborhoods with high cancer mortality. Because of the spatial autocorrelation effect, some traditional statistical models assuming the independence and randomness of variables are not suitable for studying public health events that are attached to geography, such as the COVID-19 incidence in this study, as they may bias the estimation results [17]. Following the line of the studies examining the spatial autocorrelation effect, two commonly used spatial regression models, the spatial lag model (SLM) and the spatial error model (SEM) [18] are adopted in this study.

This study aims to identify the impact of racial segregation and testing site accessibility on the COVID-19 incidence rate in cities/towns of Massachusetts. To the authors’ knowledge, this is the first study to apply spatial analysis methods to the neighborhoods’ COVID-19 data in the U.S. The objectives are to: (1) evaluate the racial segregation of minorities, such as Hispanics, Non-Hispanic Black Americans, and Non-Hispanic Asians and socioeconomic characteristics in Massachusetts; (2) access the spatial accessibilities to testing sites across different sociodemographic groups in the study area; (3) investigate whether neighborhoods with higher COVID-19 incidence rate are positively associated with highly segregated areas for minority ethnics; (4) explore whether testing sites are well distributed for COVID-19 testing; and (5) examine the association between socioeconomic and COVID-19 incidence rate.

## 2. Study Area and Data

### 2.1. Study Area

As of 2019, Massachusetts has an estimated population of 6,547,785 people, according to the U.S. Census Bureau. Currently, Massachusetts is the fifteenth-most populous U.S. state with small fluctuation in recent years. For the racial makeup in Massachusetts, 80% of the population are White alone, 9% are Black or African American alone, 7.2% are Asia alone, and other racial populations are 3.9%. In total, 63.4% of the population are in the age group of 18 and 64, while 17% are in the age group of 65 and over [19]. There are 357 cities/towns in Massachusetts, and 4 of them are undefined areas, according to the base map provided by the U.S. Census Bureau. Massachusetts is composed of eastern, central, and western regions. Eastern Massachusetts is more urban than Western Massachusetts. Boston is the largest city, at the inmost point of Massachusetts Bay. Worcester County is usually considered to be in central Massachusetts. The center of the population of Massachusetts is in Middlesex County, in the town of Natick.

As of 20 May, Massachusetts has 88,970 confirmed cases leading in the 5th place in the U.S. and in the 3rd place for the number of deaths (6066). Of the total confirmed cases, 29.6% are White, 19.1% are Hispanic, and 9.4% are Black/African American. Within reported hospitalizations, the percentage of Non-Hispanic White increases to 48.1%. Specifically, Hispanic and Black/African Americans each compose 13.3% and 11.6%, respectively. These numbers suggest that White Americans infected with COVID-19 have a higher rate of hospitalization than that of Hispanic and Black/African Americans. Thus, an analysis of the racial and health care access disparities is needed in Massachusetts.

**Figure 1:**
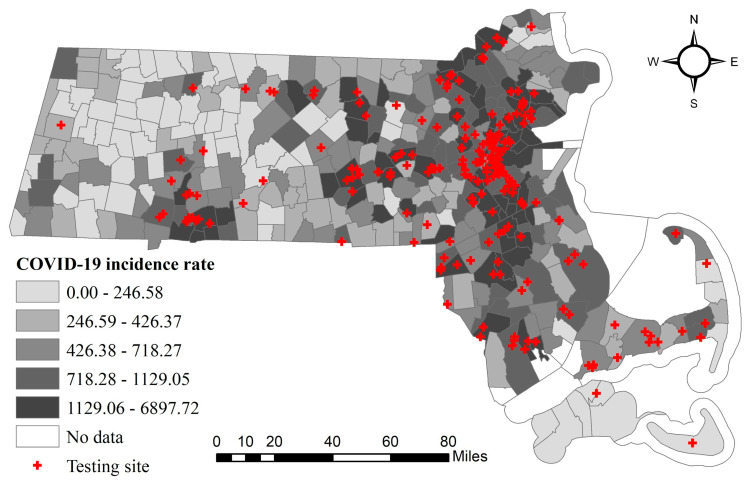
City/town-level accumulated COVID-19 incidence rate (confirmed cases/per 100,000 people) and testing sites in Massachusetts as of 20 May 2020.

On 10 March, Massachusetts declared a state of emergency, taking steps to limit the spread of COVID-19. The Department of Public Health provides not only daily and cumulative reports on Massachusetts COVID-19 cases, testing, and hospitalizations but also weekly and biweekly reports including nursing facility data, cases by city/town, residents subject to COVID-19 quarantine, and data from State facilities. The city/town COVID-19 incidence rate (cases per 100,000 people) data were recorded from 14 April. The Massachusetts government also publishes testing site locations in the state, and we geocoded the location to latitude and longitude. Figure 1 demonstrates the spatial distribution of the COVID-19 incidence rate and testing sites as of 20 May 2020. There is an extremely uneven distribution pattern: cities/towns with high incidence rates were aggregated in the Greater Boston area. In the central and west of Massachusetts, a few places are the hotspots of high incidence rates.

### 2.2. Data Preparation

This study aimed to investigate the relations between COVID-19 incidence rate and racial residential segregation, access to testing sites, and other sociodemographic and economic variables. These data were gathered from different sources. The city/town COVID-19 incidence rate data were collected from the Department of Public Health in Massachusetts. The racial residential segregation and disparities in testing site accessibilities were compiled from racial population data in cities/towns and sub-unit, census block groups. There have been many studies involving various socioeconomic and demographic factors that may impact the COVID-19 incidence rate, including but not limited to, the elderly population rate, poverty rate, overcrowding rate, and household income. We collected such variables both from the subcounty and census block group (CBG) 2018 American Community Survey (ACS) provided by the U.S. Census Bureau (https://www2.census.gov/geo/tiger/TIGER_DP/2018ACS/).

This study estimated minorities’ residential segregations, including Hispanic, Non-Hispanic Black or African American, and Non-Hispanic Asian. To simplify the name of each race, we used Black and Asian to present Non-Hispanic Black or African American and Non-Hispanic Asian in the rest of the paper. Residential segregation can be discussed in five distinct dimensions: centralization, concentration, clustering, unevenness, and isolation [20]. This study applied the isolation index to associate segregation and health outcomes. The isolation index can effectively reveal the racial differential sizes [21]. Additionally, it is different from the racial percentage, for which it is hard to determine the racial distribution disparities. The higher section of the racial isolation index indicates the same racial population has a higher chance to live as neighbors. It is a vital variable to study COVID-19 transmission patterns. Thus, this research applied the isolation index to estimate the segregation of each race. The isolation index works as follows. Assuming city/town *j* consists of *n* census block groups, the isolation index for a race within *j* can be presented as:

(1) $$R_{j}=\sum_{i=1}^{n}\frac{b_{i}}{b_{total}}\times \frac{b_{i}}{T_{i}},$$

where *i* is the *i*-th census block group in the city/town *j*; *b_i_* is the race population in *i*; *b_total_* is the total race population in *j*, and *T_i_* is the total population in *i*. The isolation index ranges from 0 to 1. A score of 0 indicates no segregation, and 1 indicates the greatest segregation. The index presents the chance of people having the same race as their neighbors.

Access to testing sites is measured by proximity, a popular method in geographic information systems (GIS), and the most influential component in health-related studies, that is, the shortest travel times to the nearest testing sites and hospitals, respectively. Both the testing site providers and primary care physicians in the hospitals are the first to contact with probable COVID-19 patients. One issue is that people may not go to the closest site for testing whether or not they are infected with COVID-19 due to various reasons, such as a long waiting time and no available providers. They may divert to the secondary choice of traveling a longer distance to an alternative testing site. Another issue may be the omission of spatial accessibility accounting for both supply and demand. Due to the unknown capacity of testing sites and hospitals for COVID-19, such two travel times are still acceptable to estimate access to COVID-19 related health care services. We used the population-weighted centroids of 357 cities/towns calibrated from the 2010 census block level data to represent the location of the population in Massachusetts. Compared to the geographic centroids, the population-weighted centroids have better accuracy, particularly in rural or peripheral suburban areas [22], and are thus commonly used. The location information of testing sites was first downloaded from the Massachusetts Government website and then geocoded by Google Maps. We also geocoded the location of hospitals extracted from COVID-19 Weekly Public Health Report. There are 228 testing sites and 76 hospitals in Massachusetts. Note that there might be some people crossing the state’s border to neighboring states or some other people outside of the state coming for testing. We did not consider these two cases. The travel times to testing sites and hospitals were estimated through the Google Maps Distance Matrix API, as it represents the dynamic traffic conditions and routing rules [23], which are not captured by the simplified Euclidean or road network-based distance accounting for the speed limits. In addition, we calculated the weighted average travel times based on the population across four demographic groups.

Overall, the data used in this study are in five categories: racial segregation, accessibility, demographics, economics, and transportation. In terms of demographic category, elderly population rate (percent of 65 years and over), overcrowding rate (percent of more than 1 occupant per room), and education attainment were estimated. The economic variables include poverty rate, household income, and income inequality. Transportation includes road density and public transit rate (percentage of workers who go to work by public transportation). Table 1 presents the category, variable name, definition, data source, and summary statistics of each variable.

## 3. Methodology

In this study, we first used Moran’s I index to test the spatial autocorrelation pattern of the city/town-level COVID-19 incidence rate in Massachusetts. Then, two classical spatial autoregressive models, including the spatial lag model (SLM) and the spatial error model (SEM), were adopted to determine the factors that influence the COVID-19 incidence rate.

### 3.1. Spatial Autocorrelation Test

Compared with geographical events that are far apart from each other, neighboring geographical events tend to share similar conditions, which makes them more closely related to each other than distant events [17]. This phenomenon is referred to as spatial autocorrelation or spatial dependency, which indicates whether the distribution of data depends on geographical locations [24]. The spatial autocorrelation effect is a common phenomenon. For instance, neighboring properties tend to have similar prices because they share common geographic locations, urban amenities, transport facilities, and so on. Therefore, house prices are usually found to be positively autocorrelated in space. If data at adjacent locations are similar to each other, the spatial autocorrelation effect is regarded as positive. If data at adjacent locations differ greatly from each other, the spatial autocorrelation effect is negative. If the distribution of data is irrelevant to geographical locations, there is no obvious spatial autocorrelation effect. A commonly used indicator to identify the spatial autocorrelation effect is Moran’s I index [25], which is formulated as:

(2) $$x=\frac{n\sum_{i=1}^{n}\sum_{j=1}^{n}w_{ij}\left(x_{i}-\bar{x}\right)\left(x_{j}-\bar{x}\right)}{\left(\sum_{i=1}^{n}\sum_{j=1}^{n}w_{ij}\right)\sum_{i=1}^{n}{\left(x_{i}-\bar{x}\right)}^{2}}$$

where *x_i_* and *x_j_* mean the attribute of *i*-th and *j*-th spatial unit (the COVID-19 incidence rate of a city/town in this study), $\bar{x}$ indicates the average of all the attributes, *n* means the number of spatial units, and *w_ij_* is a member of the spatial weight matrix *W,* which represents the spatial relationship between spatial unit *i* and *j*. In this research, we adopted the frequently-used rook method to construct the weight matrix, i.e., if spatial unit *i* and *j* share a common border, then *w_ij_* = 1, otherwise, *w_ij_* = 0 [26].

The value of Moran’s I index ranges between -1 and 1: value less than 0 means a negative spatial autocorrelation effect, and the smaller the value is, the stronger the negative spatial autocorrelation effect is; a value greater than 0 indicates a positive spatial autocorrelation effect, and the larger the value is, the stronger the positive spatial autocorrelation effect is; and a value equal to 0 means no spatial autocorrelation effect. Spatial autocorrelation analysis is based on classical statistics to measure the spatial dependence of events in adjacent locations. The statistical significance of Moran’s Index can be evaluated by the widely used Z-value, which is formulated as:

(3) $$Z=\frac{I-E\left(I\right)}{\sqrt{Var\left(I\right)}}$$

where *I* represents the Moran’s I index, *E*(*I*) refers to the mean value of Moran’s I index, and *Var*(*I*) refers to the variation of Moran’s I index. If the *Z*-value is out of the range of −1.96 and 1.96, there is a spatial autocorrelation effect at a confidence level of 95%.

### 3.2. Spatial Regression Model

Spatial data usually present a certain degree of positive spatial autocorrelation, i.e., attributes of adjacent geographical events tend to be more similar than attributes of geographical events that are further away from each other [17]. The existence of spatial autocorrelation effect violates the basic independent identical distribution (IID) assumption of classical regression models, i.e., observations are independent of one another. The significance of estimates will be overestimated if this effect is not taken into account [18]. In this study, we applied two common spatial regression models to tackle the spatial autocorrelation effect, including the spatial lag model (SLM) and the spatial error model (SEM). The SLM and SEM account for the spatial autocorrelation effect in different ways: in the SLM, the dependent variable at a location is influenced by dependent variables of neighboring locations. However, in the SEM, the spatial autocorrelation effect is represented through the remainder terms, i.e., the error at a location is affected by errors from neighboring locations.

The SLM can be formulated in terms of COVID-19 incidence rate:

(4) $$\gamma_{i}=\beta_{0}+\sum_{j}\beta_{j}X_{ij}+\rho W\gamma_{i}+\varepsilon_{i},$$

where *i* denotes a city/town, $\gamma_{i}$ indicates the dependent variable (COVID-19 incidence rate) of the *i*-th city/town, *X_ij_* means the *j*-th explanatory variable of city/town *i*, *β_0_* and *β_j_* are unknown parameters to be estimated which measure the association between COVID-19 incidence rate and covariates ceteris paribus, $W\gamma_{i}$ is the spatial lag variable in which *W* is a spatial weight matrix, ρ is the spatial autoregressive coefficient that indicates the spatial dependence of the explained variable, and $\varepsilon_{i}$ is a random error term.

The SEM is formulated as:

(5) $$\gamma_{i}=\beta_{0}+\sum_{j}\beta_{j}X_{ij}+{\left(I-\lambda W\right)}^{-1}\mu_{i},$$

where *I* is an identity matrix, λ is the spatial autoregressive coefficient that represents the spatial dependence of residuals, and $\mu_{i}$ is a normally distributed error term. The remaining symbols have the same meaning as those in Formula (4).

As shown in Table 1, the covariates have different units of measurement and large disparities in magnitude; we standardized these variables to have a mean of 0 and a variance of 1. This could make the parameter estimates independent of units and easy to compare.

## 4. Results

### 4.1. Exploratory Analysis

**Figure 2:**
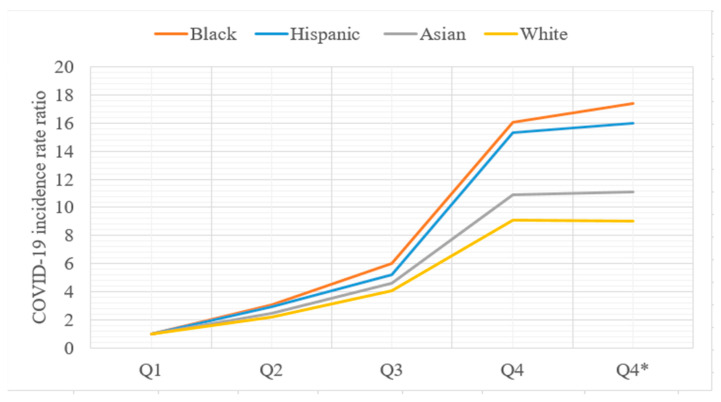
Variation of COVID-19 incidence rate across racial population in city/town, M.A. The COVID-19 incidence rate ratio represents the ratio of the COVID-19 incidence rate in each quantile compared to the first quantile.

The COVID-19 incidence rate varies across different sociodemographic groups at the city/town level. As shown in Table 2, the Non-Hispanic Black population had the lowest mean value of 2.75% ranging from 0 to 43.95%, followed by Asian and Hispanic populations with mean values of 3.6% and 4.83%. The White population had the highest mean value of 91.06% ranging from 39.27% to 100%, reflecting the dominance of White people in Massachusetts. These four groups experienced very similar COVID-19 incidence rates across the first three quantiles, but the rate differences enlarged in the fourth quantile. Using the first quantile as a reference, Figure 2 shows the variation of the COVID-19 incidence rate ratio of each quantile with that of the first quantile. All four groups initially exhibited a smooth increase with minor differences, but soon after, the ratio dramatically increased to a range of 9 to 16. Due to a large amount of confirmed COVID-19 cases in Boston city, we recalculated the COVID-19 incidence rate in the fourth quantile outside of Boston city in Table 2 and Figure 2. The rate ratios of Asian and White were consistent with those in the entire study area, while Non-Hispanic Black and Hispanic groups exhibited a small increase in incidence rate, which corroborate the concentrated segregation indices of Non-Hispanic Black and Hispanic in Boston city in Figure 2.

**Figure 3:**
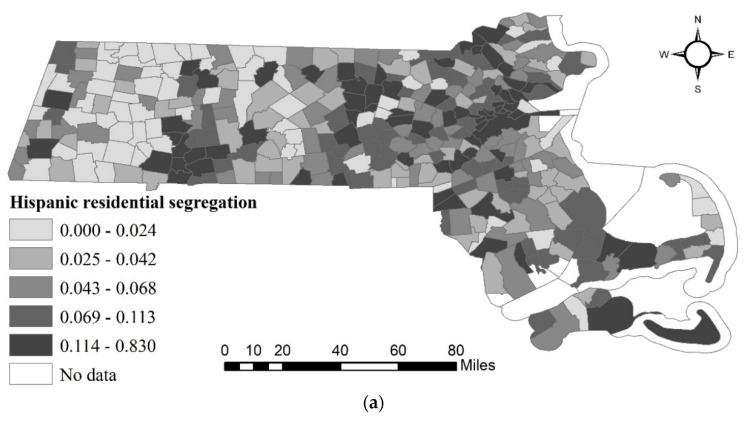

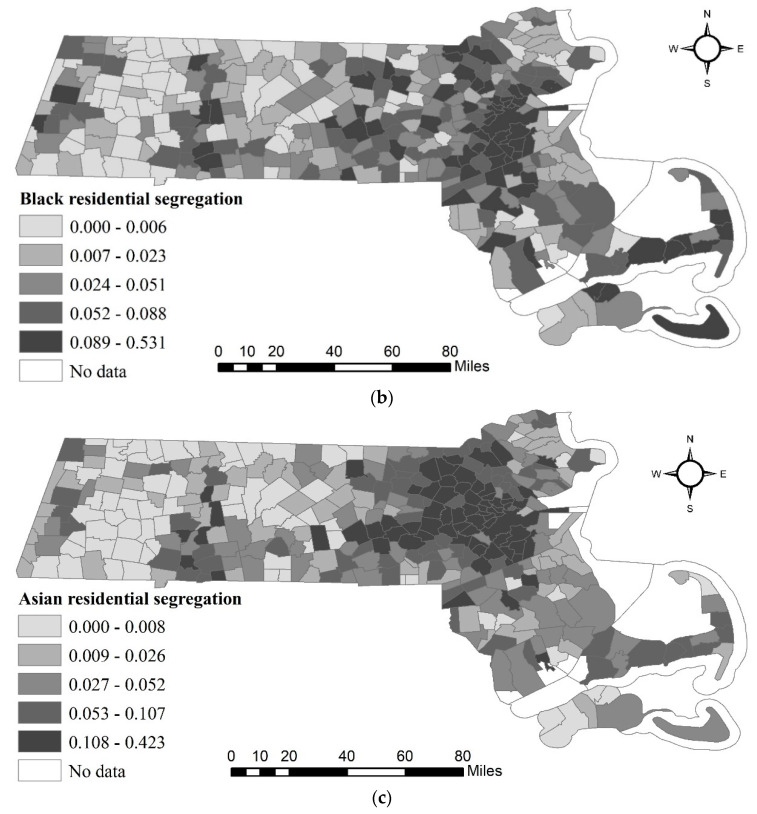
Racial segregation index of Hispanic, Non-Hispanic Black, and Non-Hispanic Asian: (**a**) Hispanic residential segregation in Massachusetts cities/towns; (**b**) Non-Hispanic Black residential segregation in Massachusetts cities/towns; (**c**) Asian residential segregation in Massachusetts cities/towns.

Racial residential segregation was estimated by the isolation index described in Section 2. Figure 3 illustrates the segregations of the minorities in Massachusetts, including Hispanic, Non-Hispanic Black, and Asian. Higher values indicate a greater possibility that people of the same race are living as neighbors. As shown in Figure 3, Hispanic and Non-Hispanic Blacks in Boston had higher segregation indexes than Asians, as Asians were more likely to live together in Middlesex and Norfolk. Middlesex County is amongst the top 25 most populated counties with the highest household income.

To quantify the health disparities across different racial groups, the weighted travel times to testing sites and hospitals are illustrated in the last two columns of Table 2. The Non-Hispanic Black group had the lowest weighted travel time of 5.69 min to the testing sites, followed by the Hispanic, Asian, and White groups. Access to hospitals showed similar results, with a minor difference. The Hispanic group surpassed the Non-Hispanic Black with the lowest travel time of 8.88 min. Compared with the other three groups, the White group had the longest travel times of 7.82 and 11.21 min to testing sites and hospitals, respectively, which is consistent with the lowest COVID-19 incidence rate in more concentrated quantiles. In addition, the travel times to testing sites and hospitals were both in acceptable ranges less than 15 min across each group. However, the travel times to testing sites were almost 3 min less than the travel times to hospitals, which revealed the condition of accessible health care services related to COVID-19 in Massachusetts.

### 4.2. Results of Spatial Regression Analysis

We conducted a Moran’s I test to examine the spatial effects. The corresponding result implied that the spatial distribution of city/town-level COVID-19 incidence rate offered strong evidence of spatial autocorrelation with Moran’s I equals to 0.436, which was positive and significantly different from expected Moran’s I of −0.003 (*p*-value < 0.001, Z-score = 13.196). The positive sign implied the existence of the spatial adjacency effect. The COVID-19 incidence rate of a city/town affects that of other nearby cities/towns, partially because adjacent cities/towns share similar attributes. The *p*-value was considerably lower than 1%, indicating that the existence of spatial autocorrelation in the COVID-19 incidence rate was statistically significant.

After correlation analysis (correlation coefficients < 0.6), among the 12 candidate explanatory variables, 9 variables were selected to be included in the final models. These variables were the percentage of elderly people (65 years and over), percent of more than 1 occupant per room, poverty rate, income inequality, road density, and test site accessibility. Table 3 shows the results of the two spatial regression models. For comparison, the classic OLS model was also calibrated, and results were listed.

Three metrics were used to compare the performances of the OLS, SLM, and SEM: log-likelihood at convergence (log-likelihood), Akaike information criterion (AIC), and R-squared (R^2^). For log-likelihood and R^2^, a higher value means better performance; for AIC, a lower value represents better performance. The results of the model performances suggested the following. First, two spatial models could better fit the observations than OLS could: the AIC of the OLS (5263.18) was much larger than in the SLM (5213.96) and SEM (5210.48), while the log-likelihood and R^2^ of the OLS (−2621.59, 0.579) were smaller than those of the SLM (−2596.979, 0.654) and SEM (−2594.24, 0.657). Second, the performance of the SEM was slightly better than that of the SLM: the log-likelihood and R^2^ of the SEM were larger than those of the SLM, while the AIC of the SEM was smaller than that of the SLM. The highest R^2^ was achieved by SEM (0.656657), which explains 65.67% of the total variations of COVID-19 incidence rates.

A tenet of regression is that residuals should be independent of each other, that is, they should be randomly distributed in space. Furthermore, the degree to which residuals are autocorrelated in space is another important indicator to judge the performance of regression models. The results showed that the residuals of the OLS revealed a strong positive autocorrelation, while the residual autocorrelations of the SLM and SEM were eliminated, and the residual autocorrelation of the SEM was eliminated more thoroughly than that of SLM (the Z-score of SEM (0.140) is smaller than that of SLM (1.017)).

According to the above-mentioned results, by incorporating spatial dependence, the two spatial regression models improved the performance of OLS in modeling the COVID-19 incidence rate in Massachusetts. Additionally, the SEM outperformed the SLM, meaning that the spatial spillover effect in the data was mainly reflected in the residuals. Therefore, to account for the spatial autocorrelation effect, the SEM specification is the more appropriate choice in this research. 

The results of the three models demonstrate that segregations of Hispanic and Non-Hispanic Black were significantly positively associated with the COVID-19 incidence rate, while segregation of Asians had a negative and nonsignificant influence on the COVID-19 incidence rate. As for the results of SEM, a one-point increase in the segregation of Hispanic and segregation of Non-Hispanic Black was, respectively, associated with a 215.677-point and 47.236-point increase in the COVID-19 incidence rate, while a one-point increase in the segregation of Asian was associated with a 47.194 decrease in the COVID-19 incidence rate. The accessibility of the test site had a negative impact on the COVID-19 incidence rate, and this impact was statistically significant in the results of OLS and SEM. For every one-point increase in test site accessibility, the COVID-19 incidence rate decreased by 75.207, as shown by the results of the SEM.

As demonstrated by the results, the rate of the population older than 65 was negatively associated with the COVID-19 incidence rate, and the coefficient was low, while the results of two spatial regression models showed that this relationship was positive. However, the results of the three models were not statistically significantly correlated with the incidence rate. As to the rate of households with more than 1 occupant per room, the results were all significantly positive. Specifically, the result of SEM indicated that a one-point increase in the rate of households with more than 1 occupant per room was associated with a 157.385-point increase in COVID-19 incidence rate. With a parameter estimate of −66.217 (SEM), the rate of the population below the poverty level had a significantly negative influence on the COVID-19 incidence rate. The association between this variable and the COVID-19 incidence rate was also negative in OLS and SLM, although the relationship had no statistical significance in the result of SLM. The results of the three models demonstrated that income inequality had a nonsignificant and negative impact on the COVID-19 incidence rate. Road density had a statistically significant strong and positive influence on the COVID-19 incidence rate, as demonstrated by the results of three models. A one-point increase in road density was associated with a 226.89-point increase in COVID-19 incidence rate, according to the result of SEM.

## 5. Discussion

Racial/ethnic segregation is regarded as a fundamental cause of disparities in diseases [9]. This study investigated the association between racial segregation and the COVID-19 incidence rate in Massachusetts, particularly minority groups, such as Hispanic, Black/African American, and Asian. We found that higher Hispanic and Black/African American segregations are more likely to be associated with a higher COVID-19 incidence rate. The areas where many Black people reside are in poor areas that are characterized by high housing densities [27]. As revealed in the regression model, a higher percentage of more than 1 occupant per room and a higher poverty rate is also significantly associated with the incidence rate. The higher observed incidence and severity in minority groups may also be associated with socioeconomic, cultural, or lifestyle factors, genetic predisposition, or pathophysiological differences in susceptibility or response to infection [28]. Furthermore, Bhala [2] proposed that the role of culture, including multigenerational households, and variation in social interactions both play important roles in the increased risk of contracting COVID-19. Minorities also contribute to heightened exposure to COVID-19 because they are more likely to work in industries that have remained open during the non-essential business closures [5].

Racial disparities in medical care treatments and outcomes are pervasive [29]. In Massachusetts, Black and Hispanic residents were less likely to be insured than White residents, and they were more likely to report fair or poor health than Whites [30]. Severe disease increases the chance of being infected by COVID-19. For example, Yang et al. [31] conducted a meta-analysis of eight studies, including 46,248 patients with laboratory-confirmed cases of COVID-19. This indicated that those with the most severe disease were more likely to have hypertension, respiratory disease, and cardiovascular disease. Moreover, other studies found that obesity and smoking were associated with increased risks [32,33]. In Italy, higher risks have also been reported in men than in women, which could be partly due to their higher smoking rates and subsequent comorbidities [34]. This pandemic presents a window of opportunity for achieving greater equity in the health care for all vulnerable populations [4].

The access to testing sites shows that the Non-Hispanic Black population has the shortest drive time of 5.69 min to testing sites, followed by Hispanic, Asian, and Non-Hispanic White, which is consistent with that to hospitals, with travel times of 9.22, 8.88, 9.96, and 11.21 min. All travel times to two kinds of sites for COVID-19 testing are acceptable. Such findings show some discrepancies addressed in previous studies. Yancy [27] demonstrated that African Americans have higher rates of COVID-19 infection and death. Such a finding was not displayed in Massachusetts, as it has a lower Non-Hispanic Black population of 9% with 9.4% COVID-19 confirmed cases, while the Non-Hispanic White is predominant (comprising 80% of the population) with 29.6% confirmed cases. Another study stated that COVID-19 testing centers are more likely to be established in the well-off suburbs, where the White population is more predominant, than in low-income Non-Hispanic Black dominant neighborhoods [11]. Most testing sites and hospitals of Massachusetts are distributed in neighborhoods dominated by minorities, as they tend to live for more job opportunities and accessible public transportation. The longer travel time of the Non-Hispanic White population to access testing sites and hospitals may be attributable to their preference of living far from cities for spacious houses. Although easy access to testing sites might not be the only factor that influences the COVID-19 incidence rate, it is undeniably a crucial component in identifying potential carriers of COVID-19. Note that in this study, we assumed car-driving is the only mode of transportation. Our omission of other modes of transportation, such as public transit in well-developed regions, may present a bias in travel times for testing. 

**Figure 4:**
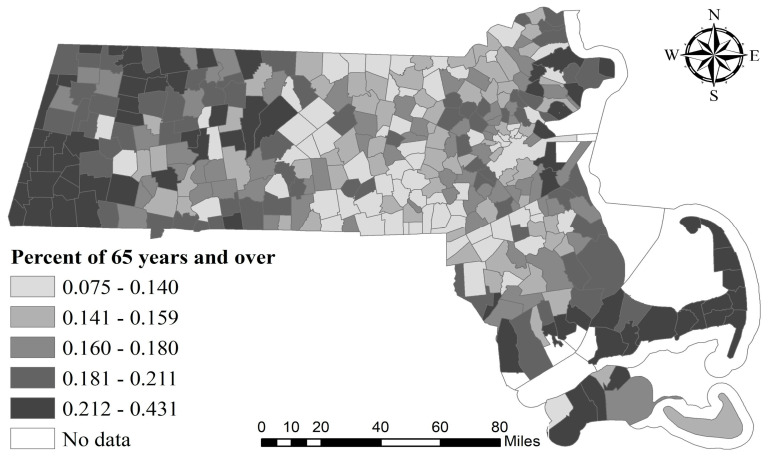
Geographical distribution of percentage of population 65 years and over.

Previous studies have demonstrated that people over 65 are most at risk for COVID-19 infection [35]. However, our study suggested that the relationship between COVID-19 incidence rate and the proportion of older people had no statistical significance. This may be attributable to the geographical distribution of the senior citizens in Massachusetts. As shown in Figure 4, cities/towns with high percentages of the elderly population are mainly located in the west and eastern coastal areas, which are far from the Greater Boston area.

**Figure 5:**
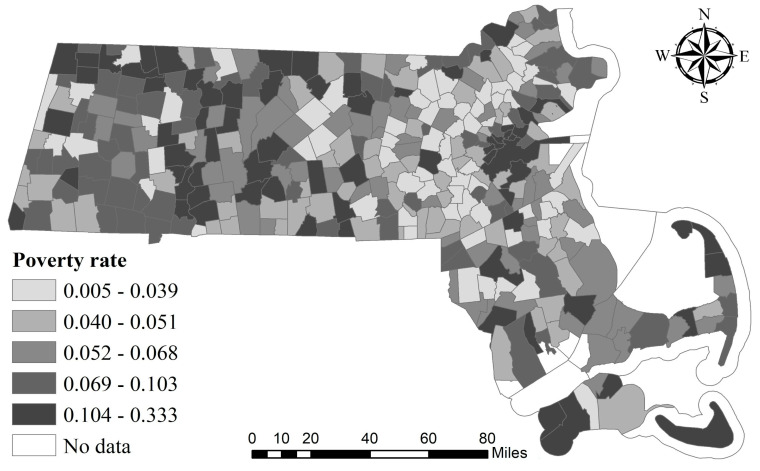
Geographical distribution of poverty rate.

Results of SEM suggested a strongly significant and positive relationship between COVID-19 incidence rate and the percentage of occupied housing units with more than 1 occupant per room. The act of quarantining individuals is often among the first responses against the prevention of infectious diseases. However, it is estimated that 44% of secondary cases were infected during the index cases’ pre-symptomatic stage in settings with substantial household clustering and quarantine outside the home [36]. In lower income households where several indviduals share the same room, the possibility of viral transmission may increase. This study has validated this hypothesis by showing that a higher percentage of more than 1 occupant per room and higher poverty rates are significantly associated with the incidence rate. The results of SEM also suggested a strongly significant and negative impact of the poverty rate on the COVID-19 incidence rate within Massachusetts. At first glance, this result is counterintuitive, since those in poverty are generally at risk of losing their health insurance coverage, which makes them vulnerable in the face of emergent epidemics such as COVID-19. However, this phenomenon can be explained by the fact that most cities/towns with high poverty rates are located in peripheral areas, which are remote from high COVID-19 incidence regions, as can be seen from Figure 5. Interestingly, the road density is significantly positively associated with the COVID-19 incidence rate. This may be because cities/towns with higher road densities typically have more frequent human activities, which could provide conditions for disease transmission.

## 6. Conclusions

The geographic disparity of the COVID-19 incidence rate has been recognized in previous studies [37,38,39]. However, most research was conducted at or above the county level, which prevents us from observing how the COVID-19 incidence rate interacts with a multitude of factors at a finer spatial scale. This study was able to analyze how the COVID-19 incidence rate was associated with racial and health accessibility disparities at a finer scale, after controlling for the possible influences of a set of demographics, economic, and transportation factors. The study was conducted at the city/town level in the State of Massachusetts, which is one of the states hit hardest by the pandemic.

The classic OLS regression model is unable to deal with the spatial autocorrelation effect. Therefore, we conducted a further spatial regression analysis based on two models, i.e., SLM and SEM, to obtain a more robust estimation of the significances and directions of the influences of racial and health accessibility disparities, demographic, economic, and transportation characteristics on COVID-19 incidence rate. Results suggest that residential segregations of Hispanic and Non-Hispanic Black/African Americans are associated with an increased risk of COVID-19 infection. Similarly, road density and percent of more than 1 occupant per room have statistically significant and positive impacts on the COVID-19 incidence rate. However, test site accessibility and poverty rate are related to a decreased risk of COVID-19 infection.

The empirical findings shown in this paper can provide helpful insights and guidance for policymakers to develop public health strategies to contain COVID-19 transmissions within Massachusetts. Most importantly, political action is needed to resolve long-standing societal inequalities, addressing the injustices of public health, and tackling the COVID-19 pandemic [2,40]. Public health is complicated and social reengineering is complex, but a change of this magnitude does not happen without a new resolve [27]. Due to the overwhelming influence that road density and overcrowding have on COVID-19 transmission, social distancing or stay-at-home policies must be vigorously reinforced. As the condition of COVID-19 is still going on and evolving quickly in the United States, it is vital to explore the spatial patterns in small units, such as neighborhoods. The finer the geographic scale, the more detailed findings will be discovered. The research framework and implications proposed in this study can further serve as a reference for other states. This will allow individual states to better understand COVID-19 transmission in the context of their unique geospatial factors, such as racial and socioeconomic distributions as well as travel infrastructure.

There are several limitations and potentials in this study. Neighborhood socioeconomic deprivation may affect potential COVID-19 detection bias, especially in minority communities. Although this research found that Hispanic and Non-Hispanic Black people had the best accessibility to testing sites, low-income families without private vehicles still face challenges in accessing testing sites [41]. Thus, assuming private vehicles as the only transportation mode might be problematic for cities where the public transit service coverage is high. Meanwhile, in Dr. Fareed’s research, he found that Non-Hispanic Black and Hispanics have much lower rates of health information seeking and confidence in accessing health information than Whites [42], which may lead to their lack of COVID-19 knowledge and information regarding the distribution of testing sites. Moreover, Hispanic and Non-Hispanic Blacks tend to suspect that they were currently infected, which may raise the potential for asymptomatic infection [43]. Therefore, differential self-assessments of the likelihood of current infection may result in differential testing behavior, causing the bias of incidence rates in the area. This research focuses on the city-level variables in the absence of individual-level data. If individual socioeconomic and health records data were readily available, they would provide further insight into the assessment of the relationship between socioeconomic status, health condition (i.e., chronic disease), and disease incidence. This in turn would enable the effective distribution of limited vaccine supplies by providing policymakers with insight into which populations to prioritize (i.e., people who are 65 years and older). 

## Figures and Tables

**Table 1:** Definition, sources, and summary statistics of explanatory variables used in this study.

Category	Variable Name	Definition	Source	Mean	Std. Dev.	Min	Max
Race segregation	(1) Segregation of Hispanic	Isolation index of Hispanic	American Community Survey 2018	0.87	0.12	0	1
	(2) Segregation of Non-Hispanic Black/African American	Isolation index of Non-Hispanic Black/African American		0.06	0.07	0	0.53
	(3) Segregation of Non-Hispanic Asian	Isolation index of Asian		0.06	0.07	0	0.42
Accessibility	(4) Travel time to testing sites	The time that is required to drive from the population-weighted centroid of each city/town to the nearest testing sites (minute)	Google Maps Distance Matrix API	12.67	9.27	1	57
	(5) Travel time to hospitals	The time that is required to drive from the population-weighted centroid of each city/town to the nearest hospitals (minute)		15.68	8.60	1	61
Demographics	(6) Percent of 65 years and over	Percentage of the population over 65 years old (%)	American Community Survey 2018	18.22	5.83	7.50	43.07
	(7) Percent of more than 1 occupant per room	Percentage of occupied housing units with more than 1 occupant per room (%)		1.18	1.24	0	9.28
	(8) Educational attainment	Percentage of population (25 years and over) with an education level of no less than bachelor’s degree (%)		42.94	16.12	11.31	84.73
Economic	(9) Poverty rate	Percentage of population with an income in the past 12 months below the poverty level (%)		7.37	4.76	0.54	33.23
	(10) Household income	Median household income in the past 12 months (dollar)		85,700.6	28,662.39	0	204,018
	(11) Income inequality	Gini index of household income		0.43	0.05	0.31	0.57
Transportation	(12) Public transit	Percentage of workers who go to work by public transportation (%)		4.13	5.96	0	33.64
	(13) Road density	The total length of primary and secondary roads in a city/town is divided by the area of the city/town (per km)	U.S. Census Bureau TIGER/Line	2.87	2.31	0	13.73

**Table 2:** COVID-19 incidence rate and health access across racial population in city/town level.

Race	Percentage of Population (%)			COVID-19 Incidence Rate (Confirmed Cases Per 100,000)					Weighted Travel Times (min)	
	Mean	Min	Max	Q1	Q2	Q3	Q4	Q4*	Testing Site	Hospital
Non-Hispanic Black	2.75	0.00	43.95	1.35	4.19	8.12	21.74	23.55	5.69	9.22
Hispanic	4.83	0.00	79.07	1.48	4.37	7.78	22.73	23.72	5.92	8.88
Asian	3.60	0.00	29.90	1.83	4.51	8.41	19.95	20.34	6.19	9.96
Non-Hispanic White	91.06	39.27	100.0	1.95	4.33	7.91	17.74	17.57	7.82	11.21

Note: Q4* represents the fourth quantile of COVID-19 incidence rate outside of Boston city.

**Table 3:** Results of regression models at town/city level.

Variable	OLS	SLM	SEM
ρ (spatial lag coefficient)	-	0.433 *** (0.050)	-
λ (spatial error coefficient)	-	-	0.521 *** (0.059)
Constant	728.148 *** (23.496)	413.507 *** (42.601)	718.326 *** (43.502)
Segregation of Hispanic	242.532 *** (36.383)	211.309 *** (32.708)	215.677 *** (34.596)
Segregation of Non-Hispanic Black	112.717 *** (29.986)	75.039 *** (26.837)	47.236 * (27.690)
Segregation of Non-Hispanic Asian	−27.229 (32.422)	−37.502 (28.965)	−47.194 (31.288)
Test site accessibility	−64.349 ** (29.140)	−37.318 (26.405)	−75.207 ** (37.491)
Percent of 65 years and over	−0.359 (28.820)	12.8947 (25.741)	33.510 (32.452)
Percent of more than 1 occupant per room	155.081 *** (30.420)	153.784 *** (27.250)	157.385 *** (27.722)
Poverty rate	−93.610 *** (32.056)	−39.326 (29.119)	−66.217 ** (31.659)
Income inequality	−29.444 (29.006)	−32.067 (25.936)	−17.368 (27.376)
Road density	180.444 *** (37.660)	112.369 *** (35.468)	226.89 *** (41.298)
Log-likelihood	−2621.59	−2596.979	−2594.24
AIC	5263.18	5213.96	5210.48
R^2^	0.579	0.654	0.657
Moran’s I of residuals	0.436 *** (Z-score: 13.196)	0.032 (Z-score: 1.017)	0.002 (Z-score: 0.140)

Note: * Significant at 0.1; ** significant at 0.05 level; *** significant at 0.01 level. Standard errors are in parentheses. The diagnostic test demonstrated that the multicollinearity condition number is 4.587, which indicated that there was no serious multicollinearity.
